# A Meta Analysis of Indoor Temperature Impacts on Sleep Duration, Mortality Risk, and Economic Costs in Older Adults

**DOI:** 10.1093/geroni/igaf122.3278

**Published:** 2025-12-31

**Authors:** Sam Karsky, Carina Gronlund, Peter Larson, Michaela Marincic, Dayna Johnson, Philippa Clarke, Ketlyne Sol, Konstantinos Papaefthymiou

**Affiliations:** University of Michigan, Ann Arbor, Michigan, United States; University of Michigan, Ann Arbor, Michigan, United States; University of Michigan, Ann Arbor, Michigan, United States; 3Cubed, Knoxville, Tennessee, United States; Emory University, Atlanta, Georgia, United States; University of Michigan, Ann Arbor, Michigan, United States; University of Miami, Boca Raton, Florida, United States; University of Michigan, Ann Arbor, Michigan, United States

## Abstract

Of U.S. householders aged 60+, 19% reported difficulty paying energy bills and 11% kept their homes at unhealthy indoor temperatures (IT). Extreme IT may impact sleep health. Healthy sleep is essential for daily functioning and long-term health, yet 33% of adults report short sleep duration (SSD, < 7 hours). Quantifying IT-sleep associations can inform residential weatherization and energy-efficiency programs, including insulation and high-efficiency heating/cooling system installation. We characterized the relationship between IT and SSD and the resulting health costs of temperature-associated sleep loss. We conducted a review and meta-analyses of associations between IT and SSD and also SSD and all-cause mortality. We estimated costs of increased all-cause mortality attributable to SSD in uncomfortably warm homes as the product of 4 terms: prevalence (20%, representing probability) of 6-hour (1 hour less than SSD threshold) sleep duration in older adults, the IT-SSD association, the SSD-mortality association, and a value-of-statistical-life metric, with bootstrapped 90% confidence intervals. Based on results from the literature, we assumed that increases in bedroom IT from comfortable (22 °C) to uncomfortable (30 °C) temperatures were linked to reduced sleep duration by 1 hour in older adults. Additionally, our meta-analysis of 18 studies found SSD is associated with increased all-cause mortality in older adults (pooled RR: 1.08, 95% CI: 1.01, 1.14). Sleep-attributable health costs in uncomfortably hot households were $96,590 (90% CI: $10,240, $362,000). Indoor-environment improvements, including weatherization and energy efficiency upgrades, may offset substantial sleep-related health costs, potentially informing funding mechanisms for these programs.

